# Claire Fraser, RN, MD, OMG: history of medicine in the *Outlander* novels and series

**DOI:** 10.5195/jmla.2020.932

**Published:** 2020-04-01

**Authors:** Stephen J. Greenberg

**Affiliations:** Section Head, Rare Books & Early Manuscripts, History of Medicine Division, National Library of Medicine, Bethesda, MD, stephen.greenberg@nih.gov

## Abstract

This article discusses how Claire Randall, RN, MD, the fictional protagonist of Diana Gabaldon’s widely popular *Outlander* series, uses her knowledge of twentieth century medicine in her adventures in eighteenth century Scotland, France, and America.

*Star Wars* is done. *Game of Thrones* is over. *Downton Abbey* has (possibly) emptied its last bone china teacup. But for those of us who love our long series of books, television shows, and movies, there is still *Outlander*, the adventures of Claire Beauchamp Randall Fraser, RN, MD, as she hops back and forth between the eighteenth and twentieth centuries in Scotland and America. As this article is being written, author Diana Gabaldon is putting the finishing touches on the ninth *Outlander* novel (tentatively titled *Go Tell the Bees that I Am Gone*), and STARZ Network has signed to produce two more series for television, starting the broadcast in February of 2020.

What makes this all relevant here is how the fictional character Claire Fraser, trained in the twentieth century first as a nurse and then as a physician, applies (or at least tries to apply) her modern medicine in the eighteenth century. Her results are mixed but very interesting. Her foci are obvious: antisepsis, antibiotics, anesthesia or analgesia, nutrition, and preventive medicine in general. But it will be helpful to sketch out Dr. Fraser’s biography and training to see how her creator sets the stage. (Note: There are some low-grade spoilers here, and Jamie Fraser will not be discussed very much.) There are also some minor variations between the timelines of the books and the STARZ shows, but nothing to concern us here. The biography follows the books, as Diana Gabaldon provides some useful charts.

Clare Beauchamp is born in 1918. Orphaned at the age of five, she spends a peripatetic childhood with her paternal uncle, an archaeologist specializing in Persian antiquities. In 1937, she marries Frank Randall, historian and genealogist. Both enter the British Army at the outbreak of the Second World War. Frank joins military intelligence, and Claire enters a nurse training program. Claire spends four years as an army nurse, both in field hospitals and more permanent facilities on the island of Malta. She clearly witnesses a lot, particularly as a surgical nurse. Somewhere along the way, she develops an interest and considerable expertise in herbal medicine.

In April 1946, with the war over, she takes a holiday with Frank to Inverness in the Scottish Highlands. Through the magical properties of a ring of megaliths, she is transported to Scotland in April 1743. She practices medicine as best as she can in a number of locations and situations, from battlefields in the Highlands to an abbey hospital in Paris. In the days immediately before the battle of Culloden in 1746, malnourished, pregnant, and weighed down with the knowledge that the Highland clans will be utterly smashed at the battle (Bonnie Prince Charlie and all that), Claire reluctantly agrees to return through the stones to the twentieth century. She emerges in April 1948 with an unexplained absence of two years and an equally mysterious pregnancy.

Claire and Frank emigrate to the United States, where Frank has been offered a teaching job at Harvard and the baby, Brianna, is born. When Brianna is old enough to start school in 1957, Claire enters medical school in Boston. By 1968, she is a respected surgeon and travels with Brianna to Scotland. She decides to attempt a return to the eighteenth century through the stones. After packing a specially designed cloak with all the twentieth century medical supplies she can manage, she goes through the stones and reemerges in Scotland in November 1766. As Clare Fraser, she and Jamie emigrate to America, where she practices medicine in every conceivable situation up to and including campaigns in the American Revolution. There is a lot more to the plot, but this will do as Claire’s resume.

In short, Claire is primarily a surgeon, who is used to battlefield conditions and operating with only the roughest materials. She also has a military physician’s understanding of nutrition, sanitation, infection, and epidemic disease. With her nursing experience, she has formidable organizational skills and quickly establishes field stations with triage and treatment while muskets are still firing. She is well prepared (at least at some level) to be dropped into a war zone, which is exactly what happens. Within hours of her unexpected arrival in Scotland, she is called upon to reduce a dislocated shoulder and to dress both bayonet injuries and bullet wounds…all on the same patient. Claire also benefits from the fact that she has had the full series of vaccinations that any modern soldier and most civilians would have had as a matter of course but which have would seemed almost magical in the past. Measles, tetanus, typhus, and, most importantly, smallpox are simply not a threat to her. Her matter-of-fact immunity is little short of terrifying to the Scots, and she is accused of witchcraft more than once. She is also occasionally asked to make love potions.

Whenever Claire gets a chance to settle down, she begins to practice a less dramatic form of what could be called public health medicine, concentrating on nutrition and basic sanitation. She has little luck explaining germ theory, even after she gets hold of a microscope in 1767 (entirely possible, since this is a century after Robert Hooke and Antonie van Leeuwenhoek demonstrated the possibilities of the device). Of course, Claire has considerably more understanding of what she is looking at than her eighteenth century contemporaries. Within a few months, she is showing her extended family the plasmodium that causes malaria under her microscope, which would not be identified until 1885 by Alphonse Laveran. To treat malaria, she obtains access to Jesuit’s bark (also know as a cinchona), the natural source of quinine and already a well-known therapy. But Jesuit’s bark was expensive and hard to get in backwoods North Carolina, where Claire winds up after leaving Scotland. Instead, with the advice of Native American healers, she concocts what seems to be a fairly nasty brew out of dogwood bark, which contains no quinine but does help control bouts of fever.

Claire has better success in dealing with nutritional disease, particularly scurvy. Even at the best of times, the traditional Scottish diet was not rich in vitamin C, and the lead-up to the 1745 uprising did not improve matters. During her first “visit” to the past (1743–1746), Claire tried to get the Scots to eat what greenery she could find but usually faced resistance from Highlanders who were happy to subsist on oats, milk, and meat. James Lind would not publish his treatise on the prevention of scurvy using citrus fruits until 1753 (others had written before him suggesting a link between nutrition and scurvy), but Lind’s influence built up only gradually. Claire’s success is entirely based on her professional perseverance and the gradual awareness among her extended eighteenth century family that they were at least keeping their teeth, while others around them were losing theirs. Talking about vitamins was of no use: vitamins in general were not identified until the 1880s, and vitamin C, isolated in 1912, was not positively linked to scurvy until 1928.

The most dramatic situations arise when Claire performs surgical procedures, usually as a result of battlefield injury but also in cases of difficult childbirth and even the occasional hernia. Claire’s technological challenges are the issues that face any surgeon: sterile operating conditions, anesthesia, blood loss, medical imaging, pain management, and post-operative infection. How these are addressed in the stories is uniformly ingenious (after all, Claire Fraser *IS* a fictional character, fulfilling the dictates of a very imaginative author), but at no point is credulity really overstretched.

Sterile conditions are the first issue Claire needs to face. She spends a lot of time boiling things and pouring brandy and raw whiskey over her surgical instruments. Later, when she is more settled, she distills pure alcohol, which patients, neighbors, and seemingly everyone else want to steal and drink. She and her “team” wear masks when they are available. When preparing for battle, she sterilizes sutures and needles in advance and carries them in bottles of alcohol. The hardest part of all this seems to be convincing her patients that all this is really necessary.

Pain management during and after surgery is complex. Claire has access to a LOT of alcohol for internal use, but a drunk patient is not always a docile one. Opium, especially as laudanum (opium dissolved in alcohol, to be taken by mouth), is usually available, but as in the case of alcohol, opiate intoxication is not the same as proper anesthesia. So Claire begins to distill ether in her surgery/laboratory on Fraser’s Ridge in the back hills of North Carolina.

Ether is tricky stuff. The simplest (read: crudest) method of synthesis involves the dehydration of raw alcohol at a temperature of 125° Celsius (257° Fahrenheit) using sulfuric acid (oil of vitriol in the eighteenth century) as a catalyst. The resulting product is effective but highly volatile and inflammable. Claire has a number of accidents involving the creation and storage of her product, with results ranging from singeing eyebrows to burning down a good-sized house. It is effective but hard to control in surgery. Lacking ventilation, proper masks, and a trained anesthesiologist to assist her, Claire nearly anesthetizes herself and her entire “team” during procedures. Historically, the first demonstration of a surgical procedure where ether was used to anesthetize to patient was famously performed at the Massachusetts General Hospital in 1846. It is only later that Claire finds out that ether was commercially available in the 1770s and was used as a preventative for seasickness (a plot point too complicated to explain here).

Two areas where Claire can find no eighteenth century solution are imaging and blood loss. There is no way to cobble up an X-ray machine, although Claire does bemoan her lack thereof, especially when she is looking for fragments of shattered, soft-lead musket balls. Somewhat odd is her disinterest in replacing blood loss. While it is certainly true that Claire could hardly have set up a blood bank and lacks the reagents to do proper blood-typing (first codified by Karl Landsteiner in 1900, with much later research), she could have watched for blood agglutination in test samples, which is how Landsteiner first made his breakthrough. In the seventeenth century, researchers such as Richard Lower in England and Jean-Baptiste Denys in France transfused blood among animals, and between animals and humans with mixed results(!), but with Claire’s knowledge of what to look for, she could, in some cases at least, have worked something out. Maybe in the next book? After all, this is a physician who synthesizes her own penicillin.

With her surgical experience in the Second World War preceding her Scottish experiences, Claire is only too aware of the dangers of wound infection. Penicillin was not new in 1946; its antibiotic properties had been noted and recorded in 1928, but mass production methods were not really perfected by Howard Florey and his team until 1942. When Claire went through the stones in 1946, penicillin was just becoming widely available. In Scotland of the 1740s, Claire is limited to topical ointments of garlic and witch hazel, which she later supplements with concoctions of raw honey, which does contain recognized antibiotic properties. However, when Claire returns to Scotland the second time, from 1968 to 1766, she is much better prepared. She brings what medical and surgical supplies she can hide, including suture needles, syringes, and penicillin in tablet form. But this small stock is quickly exhausted, and soon she tries to make more.

Moldy bread is her first source, although she readily admits that she has no real way of judging which mold is the “right” mold. Her attempts to culture promising spores are constantly interrupted by all sorts of hazards, from house cats to house fires. Her eventual medicinal broths are taken by mouth. Claire worries about dosage and side effects, but she carries on. The patients tend to do her bidding; informed consent was not much of a thing in the colonies in the 1760s, and Fraser’s Ridge is not part of the Research Triangle. But the patients do seem to recover.

Later, Claire moves on to making penicillin from Roquefort cheese (to say where she obtains the cheese is another spoiler that will not be disclosed here). This is an interesting solution to the problem. The mold that produces the characteristic veining in the cheese is a subspecies of *penicillium* and ultimately derives from bread mold, but research demonstrates that *P. roqueforti* does not produce penicillin [1]. It does, however, have other infection-preventing proteins, so perhaps it couldn’t hurt.

Claire Fraser has other interesting medical adventures in the eighteenth and twentieth centuries, including the use of acupuncture to treat seasickness, which she learns from a Chinese practitioner in eighteenth century Scotland who is exiled from his homeland. But overall, the point is made: Claire is creative, resourceful, tough-minded, independent, loyal, totally devoted to the practice of healing, and entirely without guile or pretension. Her creator has given us an admirable character backed with some very solid historical medical problems and solutions of the best kind: imaginative to the point of the incredulous, but still entirely feasible, every time.

**Stephen J. Greenberg, MSLS, PhD, AHIP**, stephen.greenberg@nih.gov, Section Head, Rare Books & Early Manuscripts, History of Medicine Division, National Library of Medicine, Bethesda, MD

## References

[1] Wilkowske HH, Krienke WA (1954). Assay of various mold-ripened cheeses for antibiotic activity. J Dairy Sci.

